# The burden of underweight and overweight among women in Addis Ababa, Ethiopia

**DOI:** 10.1186/1471-2458-14-1126

**Published:** 2014-11-01

**Authors:** Yibeltal Tebekaw, Charles Teller, Uriyoán Colón-Ramos

**Affiliations:** World Health Organization, Juba, Republic of South Sudan; Department of Global Health, Milken Institute School of Public Health, George Washington University, Washington, DC USA; Lebu Area, Nifas Silk Lafto, P.O. Box 16536, Addis Ababa, Ethiopia

**Keywords:** Underweight, Overweight, Obesity, Women

## Abstract

**Background:**

Obesity and overweight are rising worldwide while underweight rates persist in low-income countries. The aim of this study was to examine changes in the prevalence of underweight and overweight/obesity among non-pregnant women aged 15-49 years, and its socio-demographic correlates in Addis Ababa, Ethiopia.

**Methods:**

The data are from 2000, 2005 and 2011 nationally representative Ethiopian Demographic and Health Surveys in Addis Ababa. The dependent variable was women’s nutritional status measured in terms of body mass index coded in binary outcomes to examine risk of being underweight (<18.5 kg/m^2^ vs. ≥18.5 kg/m^2^) or overweight/obese (>25 kg/m^2^ vs. ≤25 kg/m^2^). Logistic regression models were used to estimate the strength of associations.

**Results:**

The prevalence of overweight/obesity increased significantly by 28%; while underweight decreased by 21% between 2000 and 2011. Specifically, the prevalence of urban obesity increased by 43.3% i.e., from 3.0% to 4.3% in about 15 years. Overall, more than one-third (34.7%) of women in Addis Ababa were either under or overweight. Women’s age and proxies for high socio-economic status (i.e. household wealth quintile, educational attainment, access to improved source of drinking water, and television watching) were positively associated with being overweight. The correlates of underweight were young age and proxies for low socio-economic status (i.e. low wealth quintile, limited access to improved source of water or toilet facility).

**Conclusions:**

There is a need for policies to recognize the simultaneous public health problems of under and overnutrition, and for programs to target the distinct populations that suffer from these nutrition problems in this urban area.

## Background

Low-income countries have historically been burdened with high levels of undernutrition (stunting, wasting and underweight) and infectious diseases [1, 2]. More than 3.5 million mothers and children under-five die annually due to the underlying cause of undernutrition, and millions more are permanently disabled physically and mentally as a result of poor dietary intake in the earliest months of life [3]. Women who are underweight prior to pregnancy and who gain little weight during pregnancy are at increased risk of complications and death [1, 3]. Malnourished mothers are more likely to give birth to low birth-weight babies who face a greatly increased risk of dying in infancy. In Africa, all levels of underweight (i.e., mild, moderate and severe underweight) are highly prevalent [4, 5].

Despite this high prevalence in underweight, low-income countries are also faced with increasing rates of overweight and obesity, particularly in urban areas [2, 6, 7]. The demographic and epidemiological transitions in low income countries have been accompanied by increasing rates of urbanization, overweight and obesity and non-communicable diseases (NCD) [1–4, 8, 9]. Urbanization brings about increased consumption of refined sugars and animal fats, usually coupled with a more sedentary lifestyle; all of these promote obesity [10, 11]. Between 1980 and 2008, mean body mass index (BMI) globally increased on average by 0.4 gk/m^2^ per decade [12]. Now a days, overweight/obesity and underweight co-exist in the same community or family in low-income countries [1, 13]. The rates are increasing at alarming rates in Sub-Saharan Africa, [14] where as much as 20-50% of urban populations are estimated to be overweight or obese [15–17].

The rise in obesity prevalence represents a challenge for the health care system, which is traditionally overburdened by underweight problems arising from famine, food insecurity and infectious diseases [18], but now has to cope with obesity-related NCDs which are estimated to account for 46% of all deaths by 2030 [19].

The growing problem of obesity presents a challenge for public healthcare systems in low- and middle-income countries. The healthcare systems in these countries traditionally devote its resources to problems of underweight and infectious diseases, but as these countries experience increased economic development and urbanization, they must also learn to manage nutrition-related NCDs [20]. Urbanization brings about increases in consumption of refined sugars and animal fats, usually coupled with a more sedentary lifestyle, which promote obesity [10, 11].

Addis Ababa is an ideal setting to describe the coexisting rates of under and overnutrition. Addis Ababa is the capital and largest urban area of Ethiopia, a country that has been largely burdened by famine, food insecurity and underweight [21]. However, today NCD such as cardiovascular diseases and diabetes are the leading causes of death among adults [22]. About 31% of deaths reported from hospitals in Addis Ababa were attributed to diabetes mellitus and cardiovascular diseases [23]. The prevalence of hypertension is near 20% and 15% among healthy working men and women respectively. The obesity and overweight rates in the city are comparable to those reported in other urban areas of the Sub Saharan Africa (SSA) region, where 25.7% of women were overweight and 10.2% were obese [24], and 25% of adolescents in school were obese [25]. The trends in the increase of obesity rates have not yet been documented for this capital, and there is scarce information about the determinants of overweight. This information can help elucidate policy and program strategies to deal with the coexisting underweight and overweight problems.

## Methods

### Conceptual framework

We draw from United Nations Children’s Fund (UNICEF’s) nutrition conceptual framework to identify potential correlates of nutritional status (overweight and underweight) [26]. The framework highlights that *immediate causes* of nutritional status are diet intake and health status. The *underlying causes* of diet intake and health status in turn rest on three pillars: (1) household food security (i.e. income to buy food, access to foods); (2) maternal and caregiver practices (i.e. maternal education, inadequate or inappropriate information or education breastfeeding practices, etc.) and (3) health services and the environment (i.e., access to maternal healthcare services, exposure to media (which has been associated with being sedentary [10, 11], dwelling characteristics, access to water and sanitation). These underlying causes are in turn determined by *basic societal causes,* including cultural or socio-political characteristics that may be dictated by ethnicity, or religion, working and marital status, woman’s relationship to head of household) and economic structures (i.e. wealth or socioeconomic status). These basic societal factors may shape community and individual resources and behaviours [26]. For example, weight gain increases with parity especially in urban areas women with lower parity are more likely to have lower BMI levels [27]. In Ethiopia, health and religious beliefs have strong link [28, 29]. An in-depth analysis shows that Muslim women show better decision making power on their own health care as compared to other religious groups [30]. Another study shows that in Ethiopia, women’s decision-making autonomy has positive effect on their nutritional status [31].

### Data source

The data are from 2000, 2005 and 2011 nationally representative Ethiopian Demographic and Health Surveys (EDHS). The survey was implemented by the Ethiopian Central Statistical Agency (CSA) with the technical assistance of Inner City Fund (ICF) International through the USAID-supported MEASURE DHS project. The survey inquires about household members’ and individual characteristics using household questionnaire, woman’s questionnaire and man’s questionnaire. Individual women of reproductive aged 15-49 years were interviewed face-to-face on their background characteristics and height and weight measurements were carried out on women aged 15-49 years.

This study focused on Addis Ababa the capital of Ethiopia. EDHS employed two stage cluster sampling technique. Census enumeration areas were the sampling units for the first stage while households comprised the second stage of sampling. A fixed number of 30 households were selected for each enumeration areas. For this study, variables were obtained from the individual women's and household questionnaires. The women’s questionnaire provided information on the characteristics of the individual woman while the household questionnaire provided information on household possessions and amenities such as source of drinking water, toilet facilities and dwelling characteristics [32–34].

### Variables

#### Dependent variable

The dependent variable in this study is women’s nutritional status measured by their BMI. A cut-off point of 18.5 is used to define underweight and a BMI of 25 or above usually indicates overweight or obesity according to the WHO Expert Committee on Physical Growth [4]. Pregnant women were excluded from the study.

#### Independent variables

From the EDHS database, the following variables were identified:

*Underlying determinants*: woman’s educational attainment (no education, primary, and secondary or higher education), women’s decision-making autonomy on own healthcare, large household purchases and visits to relatives, partner’s educational status, antenatal visit and place of delivery. UNICEF’s multiple indicator cluster survey was used to define source of water and sanitation categories [35]. Source of drinking water was categorized as *improved* for those who have piped water source, public tap or standpipe, tube well or borehole, protected well or spring and rain water; and *unimproved* for those with access to water piped outside of the compound, unprotected well, unprotected spring, well or borehole, bottled water, river/dam/lake/pond/stream/canal/irrigation channel, or tanker truck. As to type of toilet facility, flush toilets, ventilated pit latrine and pit latrine with slabs were categorized as *improved* and all the rest, including pit latrine without slab, open field, composting toilet and others were grouped into ‘*unimproved’*. Exposure to media was assessed in terms of exposure to newspaper/magazine and television. Hence, each of these variables was categorized as ‘*yes’* if the respondent reads newspaper/magazine or watches television regardless of the frequency; as ‘*no’* if the respondent doesn’t read newspaper/magazine or does not watch television at all.

*Basic determinants*: women’s working status (*yes/no*), age (years), marital status (*never married, currently married, divorced, widowed, living together*), parity (*number of children ever born*), woman’s relationship to head of household (*head, wife, daughter, etc.*), sex of household head (*male/female*), age of household head (years) and religion (*Orthodox Christian, Muslim, Catholic, Protestant, Traditional*).

Wealth index was also examined in the following way: our preliminary analysis showed that at national level the majority of the study participants in Addis Ababa (94% and 98% 2005 and 2011, respectively) belonged to the highest category of the wealth quintile. However, for the purpose of this study, we developed a wealth index factor score using the principal component analysis method to regroup the study population into the wealth quintile specific to Addis Ababa. In the grouping of the wealth status, after obtaining the wealth quintiles, the 815 and 1648 sample size for 2005 and 2011 EDHS data were classified into five categories of approximately equal numbers ranging from the least advantaged (first quartile or lowest class) to the most advantaged (fifth quintile highest class). Wealth index was not included in the 2000 EDHS and hence all the analyses related to wealth quintiles in this study refer only to the 2005 and 2011 data.

#### Statistical analysis

All analyses were conducted using the Statistical Package for Social Sciences (SPSS) version 17.0. Individuals with missing values for BMI (n = 91) or any of the other covariates were excluded in the analyses. Variables including women’s decision-making autonomy on own healthcare, large household purchases and visits to relatives, partner’s educational status antenatal visit and place of delivery were excluded from the analyses for having large (50-80%) missing values.

Due to the non-proportional allocation of the sample to the different regions and to their urban and rural areas during stratification, EDHS recommends sampling weights for any analysis using EDHS data to ensure representativeness of the survey results at the national and regional level. However, in order for the survey precision in urban areas to be comparable with that in rural areas, urban areas were oversampled. The DHS also advises against use of sample weights for oversampled areas as it drastically overestimates sampling variances and confidence intervals. Since the current study was entirely based on samples from urban Addis Ababa without comparisons with other regions in the country, sample weighting was not needed in the estimation of means, proportions or ratios.

Covariates were cross-tabulated by BMI categories, and Chi-square values were used to test for significant associations. Multivariate logistic regression models were fitted for each outcome for each one of the EDHS data (six in total for years 2000, 2005, and 2011). The multivariate models included variables that statistically significantly associated with BMI levels (p-value < 0.05) in the bivariate analyses. Tests for collinearity between covariates were performed and variables (total children ever born and number of living children) that presented >10 value of variance inflation factor were not included in the final model.

## Results

In the EDHS 2000, 2005 and 2011, surveys were completed for 1996, 815 and 1648 women in Addis Ababa with urban response rates of about 98%, 96% and 94% respectively. The total number of non-pregnant women who had anthropometric measurements and included in this study was 1936, 804 and 1592 women for EDHS 2000, 2005 and 2011 respectively.

### Trends in overweight and underweight

The descriptive results in Table 1 show that the overall prevalence of overweight rose from 16.1% in 2000 to 20.6% in 2011. The prevalence of obesity increased from 3.0% in 2000 to 4.3% in 2011 or a 43.3% increase. In 2000, the prevalence of underweight (17.9%) was slightly higher than overweight/obesity (16.1%), but by 2011, the overweight/obesity prevalence surpassed the underweight prevalence (20.6% to 14.1%).

**Table 1:** **Changes over time in the prevalence of underweight, overweight and obesity of study participants in Addis Ababa, Ethiopia, 2000-2011**

BMI levels*	2000 (n = 1936)	2005 (n = 804)	2011 (n = 1592)	Percent change between 2000-2005	Percent change between 2005-2011	Percent change between 2000-2011
Underweight	17.9	16.0	14.1	-10.2	-11.9	-20.9
Overweight/obesity	16.1	17.7	20.6	9.9	16.4	28.0
Overweight	13.1	13.6	16.3	3.3	20.5	24.5
Obesity	3.0	4.1	4.3	34.7	4.1	40.2

*Underweight: <18.5 kg/m^2^, Overweight/obesity: >25 kg/m^2^, Overweight: >25 kg/m^2^, Obesity: ≥30 kg/m^2^.

Tables 2 and 3 show the cross-tabulated analyses and multiple logistic regression results respectively of socio-demographic characteristics in their association to underweight and overweight rates.

**Table 2:** **Socio-demographic characteristics and prevalence of BMI, Addis Ababa, Ethiopia, 2000-2011**

Variables	2000		2005		2011	
	***Underweight (%)***	***Overweight/obese (%)***	***Underweight (%)***	***Overweight/obese (%)***	***Underweight (%)***	***Overweight/obese (%)***
**Wealth index**						
Lowest	-	-	21.5**	7.4**	16.4*	13.0***
Low	-	-	17.8	16.6	14.0	19.1
Middle	-	-	17.8	21.5	13.7	20.7
High	-	-	15.3	17.8	15.2	23.1
Highest	-	-	6.7	24.5	9.7	29.0
**Marital status**						
Never married	21.2*	9.4*	19.3**	9.5*	18.8*	11.3*
Married	13.8	28.4	10.1	33.0	8.5	32.9
Others	14.6	15.6	14.8	20.0	9.3	28.0
**Woman's education**						
No education	15.9	12.9	14.6	12.5***	6.9**	18.5
Primary education	18.7	15.9	14.0	14.5	14.1	18.6
Secondary and higher	18.4	17.8	17.4	20.7	16.3	23.0
**Respondent currently working**						
No	21.1	14.3	19.3	14.1	14.7	18.2
Yes	14.0	18.4	11.7	22.6	13.7	22.7
**Parity**						
0	20.6*	10.3*	19.4***	9.1*	18.3*	11.6*
1-3	15.6	18.2	10.2	30.7	9.4	31.9
4+	12.1	32.7	12.5	30.8	5.1	36.2
**Number of living children**						
0	20.4**	10.3	19.4**	9.6	18.1	11.8
1-3	16.3	18.8	10.3	29.1	9.5	32.4
4+	11.0	33.9	12.0	32.6	4.9	34.4
**Source of drinking water**						
Improved source	15.7*	18.3*	14.8	21.3*	14.0	21.9***
Unimproved source	22.4	11.7	18.9	9.4	14.2	16.9
**Type of toilet facility**						
Improved source	17.2*	17.0**	14.5*	20.2**	17.7***	22.2**
Unimproved source	22.4	10.6	19.3	11.9	12.9	16.0
**Relationship to HH head**						
Head	18.6*	21.9*	13.1*	26.9*	11.7*	28.6*
Wife	13.1	29.2	9.2	34.7	7.6	31.0
Daughter	22.7	10.7	25.1	10.4	22.8	14.3
Other	16.5	8.5	14.1	8.5	14.0	12.8
**Age of HH head**						
13-24	18.5*	4.9	30.8***	10.3**	20.2***	12.8***
25-34	18.2	13.4	13.0	12.2	10.6	16.2
35-44	17.8	17.8	14.7	23.9	12.8	24.8
45-54	19.2	19.6	14.9	20.4	17.6	23.6
55+	16.4	14.5	17.8	13.4	14.0	19.3
**Religion**						
Orthodox Christian	18.2*	16.1	17.2***	18.3	14.2	21.0
Muslim	17.5	15.4	7.3	18.8	15.1	15.9
Protestant/Catholic/Traditional/Others	15.6	17.7	17.6	12.1	12.4	24.8
**Reads newspaper/magazine**						
No	17.6	14.2	13.4	14.7	11.2	19.8
Yes	18.1	16.7	18.9	16.7	16.5	21.3
**Watches television**						
No	21.4*	10.3**	19.0	13.5	12.6	9.0**
Yes	16.1	16.0	13.3	12.5	14.3	21.5
**Total/Average**	**17.9**	**16.2**	**16.0**	**17.7**	**14.1**	**20.6**

*P-value < 0.0001, **P-value < 0.01, *P-value < 0.05 for chi-square tests within categories.

**Table 3:** **Adjusted odds ratios, based on logistic regression analysis, of underweight and overweight/obesity among women of 15-49 in Addis Ababa, 2000-2011**

Variables	Underweight			Overweight		
	2000	2005	2011	2000	2005	2011
**Wealth index** *(RC = Lowest)*						
Low		0.66 [0.37, 1.18]	0.62 [0.39, 1.01]*		2.59 [1.16, 5.77]*	2.05 [1.25, 3.37]**
Middle		0.66 [0.35, 1.25]	0.54 [0.32, 0.90]*		3.03 [1.30, 7.03]*	2.19 [1.29, 3.71]**
High		0.45 [0.23, 0.89]*	0.54 [0.32, 0.93]*		3.19 [1.32, 7.72]*	3.05 [1.74, 5.35]****
Highest		0.19 [0.09, 0.44]****	0.33 [0.18, 0.59]****		3.83 [1.54, 9.53]***	4.64 [2.60, 8.28]****
**Age 5-year groups** *(RC = 15-19)*						
20-24	0.69 [0.49, 0.97]*	0.97 [0.57, 1.64]	0.88 [0.59, 1.30]	0.95 [0.57, 1.59]	1.44 [0.69, 2.99]	1.67 [0.96, 2.91]
25-29	0.69 [0.46, 1.03]	0.86 [0.46, 1.62]	0.90 [0.56, 1.45]	1.72 [1.03, 2.87]*	1.16 [0.50, 2.67]	2.58 [1.47, 4.55]**
30-49	0.56 [0.34, 0.92]*	0.71 [0.33, 1.51]	0.47 [0.26, 0.86]*	5.19 [3.02, 8.91]****	2.34 [1.02, 5.35]*	4.41 [2.45, 7.93]****
**Marital status** *(RC = Never married)*						
Married	0.83 [0.45, 1.54]	0.87 [0.28, 2.71]	0.89 [0.42, 1.90]	1.84 [0.95, 3.54]	1.17 [0.40, 3.37]	1.63 [0.89, 2.98]
Others	0.72 [0.43, 1.18]	1.06 [0.44, 2.54]	0.77 [0.40, 1.49]	1.25 [0.70, 2.25]	0.93 [0.37, 2.32]	1.37 [0.79, 2.39]
**Woman’s education** *(RC = No education)*						
Primary education	1.14 [0.80, 1.63]	0.92 [0.48, 1.77]	1.68 [0.90, 3.11]	1.61 [1.08, 2.41]*	1.67 [0.82, 3.41]	1.40 [0.91, 2.17]
Secondary and higher	1.11 [0.80, 1.54]	1.47 [0.82, 2.65]	2.10 [1.07, 4.09]*	2.36 [1.62, 3.44]****	1.95 [1.00, 3.80]*	1.35 [0.85, 2.13]
**Parity** *(RC = 0)*						
1-3	0.91 [0.57, 1.44]	0.68 [0.27, 1.67]	0.88 [0.48, 1.61]	0.60 [0.36, 0.99]*	2.21 [0.91, 5.36]	1.52 [0.93, 2.49]
4+	0.70 [0.38, 1.29]	1.20 [0.38, 3.79]	0.68 [0.24, 1.91]	0.83 [0.47, 1.47]	2.09 [0.73, 6.00]	1.31 [0.69, 2.49]
**Source of drinking water** *(RC = Improved)*						
Unimproved	1.60 [1.20, 2.12]**	0.88 [0.54, 1.42]	0.75 [0.50, 1.10]	0.65 [0.47, 0.92]*	0.61 [0.34, 1.09]	1.17 [0.81, 1.70]
**Type of toilet facility** *(RC = Improved)*						
Unimproved	1.15 [0.79, 1.67]	1.06 [0.66, 1.69]	1.32 [0.93, 1.88]*	0.79 [0.48, 1.29]	0.85 [0.50, 1.47]	0.99 [0.70, 1.41]
**Relationship to HH head** *(RC = Head)*						
Wife	0.67 [0.38, 1.18]	1.00 [0.37, 2.67]	0.65 [0.34, 1.26]	1.03 [0.62, 1.71]	0.90 [0.42, 1.91]	0.78 [0.49, 1.24]
Other	0.59 [0.35, 0.98]*	1.62 [0.75, 3.50]	1.03 [0.56, 1.88]	0.76 [0.44, 1.31]	0.49 [0.25, 0.98]*	0.56 [0.33, 0.95]*
**Age of HH head** *(RC =* *≤* *34)*						
35-44	1.17 [0.80, 1.73]	0.76 [0.40, 1.42]	1.36 [0.85, 2.18]	1.02 [0.65, 1.60]	2.20 [1.10, 4.39]*	1.29 [0.85, 1.96]
45-54	1.33 [0.89, 1.99]	0.74 [0.40, 1.38]	1.64 [1.02, 2.64]*	1.28 [0.80, 2.05]	1.98 [0.94, 4.18]	1.47 [0.94, 2.31]
55+	1.04 [0.68, 1.58]	0.80 [0.42, 1.52]	1.08 [0.65, 1.78]	1.17 [0.70, 1.96]	1.86 [0.80, 4.29]	1.76 [1.07, 2.90]*
**Religion** *(RC = Orthodox)*						
Muslim	0.93 [0.64, 1.34]	0.31 [0.14, 0.72]**				
Others	0.82 [0.54, 1.26]	0.91 [0.49, 1.67]				
**Watches television** *(RC = No)*						
Yes	0.67 [0.49, 0.92]*				1.89 [1.27, 2.81]**	2.28 [1.12, 4.65]*

*P-value < 0.05, ** < 0.01, *** < 0.001, **** < 0.0001 and no asterisk > = 0.05.

### Underweight and its correlates

**Figure 1:**
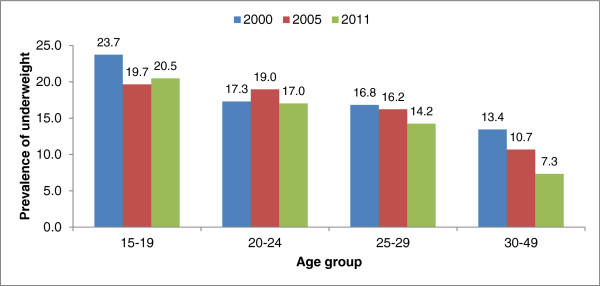
**Prevalence of underweight by age of women in Addis Ababa, Ethiopia, 2000-2011.**

The highest prevalence rates of underweight were observed among younger age (15-19), never-married, nulliparous, those without improved sources of drinking water and without access to improved toilet facility. The highest decline in the prevalence of underweight was observed in the age group of 30-49 (-45.4%) followed by those aged 25-29 years (-15.3%) (Figure 1). Prevalence of underweight decreased by 23.7% among the lowest wealth quintile households but increased by 44.7% among those of the highest wealth quintiles between 2005 and 2011. The rate of underweight decreased by 23.1% among women of the middle wealth quintile households between 2005 and 2011.

Women who belong to the highest (fifth) wealth quintile households were 81% (OR = 0.19; 95% CI: 0.09, 0.44) and 67% (OR = 0.33; 95% CI: 0.18, 0.59) less likely to be underweight in the 2005 and 2011 models respectively compared to those in the lowest wealth quintile households. Better educated women were more likely to be underweight in the 2011 model. Women in the age ranges of 30-49 were less likely to be underweight for 2000 (OR = 0.56; 95% CI: 0.34, 0.92) and 2011 (OR = 0.47; 95% CI: 0.26, 0.86) models respectively compared to younger ones (15-19). Conversely, younger women aged 15-19 years were 1.79 and 2.13 times more likely to be underweight respectively compared to those aged 30-49 years.

Women without access to improved source of drinking water and improved toilet facilities were more likely to be underweight compared to those with access to improved source of water (OR = 1.60; 95% CI: 1.20, 2.12) and toilet facilities (OR = 1.32; 95% CI: 0.93, 1.88) respectively. Women who watched television were 33% less likely to be underweight compared to those who do not watch television (OR = 0.67; 95% CI: 0.49, 0.92).

### Overweight and its correlates

**Figure 2:**
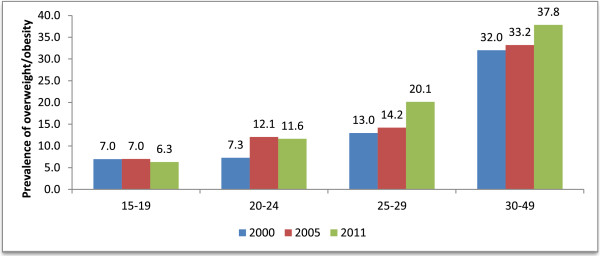
**Prevalence of overweight/obesity by age of women in Addis Ababa, Ethiopia, 2000-2011.**

The prevalence of overweight/obesity shows a progressive increment by age groups (Figure 2). In 2011, women aged 30-49 years old exhibited the highest (37%) overweight/obesity prevalence. However by comparison, the highest increment was among those 20-24 years (+60%) followed by those in the highest age group of 25-29 (+55%) between 2000 and 2011. With regards to wealth quintile, the highest prevalence rate was observed among women who belong to the household with the highest wealth quintile and those with secondary and higher education compared to the lowest wealth quintile and those with no formal education. Prevalence of overweight/obesity increased by 18.2% between 2005 and 2011 among women of the highest wealth quintile households. Compared to the increase in overweight between the 2000 and 2011 surveys (+21.3%), the prevalence of obesity alone showed an increase among those with secondary and higher education by +59.5%, from 3.7% in 2000 to 5.9% in 2011. Contrary to this, overweight/obesity prevalence decreased by 3.7% among women of the middle wealth quintile households between 2005 and 2011. The occurrence of overweight/obesity was also significantly higher among those who have access to improved drinking water source (18.3% for 2000 and 21.9% for 2011), with access to improved toilet facility (17.0% for 2000 and 22.2% for 2011), and those who watch television (16.0% for 2000 and 21.5% for 2011).

The logistic regressions show that women in the highest wealth quintile were more likely to be overweight (OR = 3.83; 95% CI: 1.54, 9.53 in 2005) and (OR = 4.64; 95% CI: 2.60, 8.28 in 2011) compared to those in the lowest wealth quintile. Educational attainment positively associated with overweight/obesity for the 2000 and 2005 models. Hence, women with secondary or higher education were over twice as likely to be overweight or obese compared to their counterparts with no education (OR = 2.36; 95% CI: 1.62, 3.44) for 2000 and (OR = 1.95; 95% CI: 1.00, 3.80) for 2005 respectively. Education did not show statistically significant association with being overweight in the 2011 model.

In the 2000 and 2011 models, women aged 30-49 years were about 2-5 times more likely to be overweight/obese compared to their youngest counterparts (15-19) (OR = 5.19) for 2000, (OR = 2.34) for 2005, and (OR = 4.41) for 2011 models, respectively. Women without access to improved source of drinking water were 35% less likely to be overweight for 2000 model compared to those with access to improved source of drinking water. In the 2005 and 2011 models, women who watch television were 1.89 (95% CI: 1.27, 2.81) and 2.28 (95% CI: 1.12, 4.65) times more likely to be overweight compared to those who do not watch television.

## Discussion and conclusion

This study provides evidence for the growing double burden of malnutrition due to increasing obesity and overweight rates among women in Addis Ababa. This is in agreement with previous studies that report increases in obesity in Africa and globally. For example, in 25 out of 33 countries in SSA had lower underweight (14.5%) and higher overweight (19.75%) adult female populations [15]; the same was reported from Bangladesh, India and Indonesia [36, 37]. While underweight prevalence has decreased in Addis, it continues to burden younger reproductive-age women who belong to the relatively lower wealth quintile households (i.e. have limited access to drinking water and toilet facilities and are heads of their households). This is in line with findings elsewhere in urban Africa including Ghana, Kenya, Malawi and Niger [9, 38].

Populations that suffer from underweight or from overweight are potentially distinct from each other in terms of socio-demographic characteristics. This is not surprising given the UNICEF framework that highlights socio-demographic characteristics that will ultimately shape diet intake and health status, producing one or the other outcome. For example, our results highlight that women in the higher wealth quintile households are more likely to be overweight and are less likely to be underweight. The wealth quintile may be a proxy for unmeasured characteristics related to improved access to health facilities and quality of care, or higher income and therefore more sedentary lifestyles and diversification of diet by including highly-caloric foods and animal sources [10, 11]. In the present study those women who watch television were less likely to be underweight but more likely to be overweight compared to those who do not watch television at all. Television-watching has been associated with excess weight gain [39] through various mechanisms; for example, it likely increases the time that viewers are sedentary [1], or the advertisements may promote unhealthy high caloric food consumption [40]. It is also associated with lower risk of underweight because it may imply television ownership which is a proxy for higher wealth or greater access to quality health care compared to those who do not watch or own television.

Similarly, women with unimproved sources of water are more likely to be underweight but less likely to be overweight, underscoring the UNICEF framework that unmeasured characteristics related to sanitation, spread of infectious diseases, and healthcare facilities can impact poor diet, health status, and lead to underweight. This is similar to findings from urban Bangladesh (37) and Saudi Arabia [41]. Improvements in water and sanitation contribute to a decrease in underweight [1]. Lack of access to safe drinking water and proper sanitation contribute to the spread of infectious diseases, which synergistically interact with undernutrition to increase mortality [26]. In addition, researchers posit that chronic exposure to pathogens and infectious diseases resulting from improper sanitation facilities can lead to stunted growth and acute underweight [42].

The association between educational status and risk of underweight is worth further discussion. According to our results, women with higher educational attainment were more likely to be underweight compared to those with no formal education in 2011. The 2005 and 2011 EDHS reports show that the percentage of underweight women among those with primary level of education was higher than those with no formal education [33, 34]. On the other hand, educational status and BMI levels associated positively in all the models. However, the strengths of associations show a steady decline from 2000 to 2011. While many studies have documented the positive association between educational status and overweight obesity [9, 16, 44, 45], evidences in other countries i.e. Brazil, Mexico, China, Poland, Ghana, South Africa show that adults with low educational status are more likely to be overweight [2, 8, 46], or show no association between education and overweight/obesity status in urban areas [43, 47]. It is worth further exploring the cause and patterns of variations in the impact of education on BMI status in the current study area.

The findings from these analyses confirm that Ethiopia’s health care system is facing the rising challenge of overweight and obesity-related NCD, particularly in the urban area of Addis Ababa. The 2011 EDHS report shows a large rural-urban gap in overweight/obesity among women aged 15-49 years, with rural areas at 2.6% vs. 14.9% in all urban areas (34). However with higher rates of population growth in urban areas due to migration, we expect this problem of obesity to increase. Globally, urban diets have been described to include higher consumption of refined grains, higher fats, more animal products, more sugar and foods prepared away from home, including highly processed, nutrient-poor foods, compared to traditional rural diets [38]. Urban lifestyle tends to be more sedentary. In Addis Ababa, 37% of the residents are rural migrants looking for improved educational and employment opportunities [18]. As they face these new environments, it is likely that the rates of overweight and obesity will continue to increase.

Ethiopia’s health care system focuses more on communicable diseases [22, 24] and the women’s component of the Federal Government’s new 2013-2015 National Nutrition Programme focuses exclusively on reducing the prevalence of chronic undernutrition [48]. Most of the nutrition-oriented resources in the predominantly rural country are allocated to address chronic food insecurity and acute and chronic undernutrition. Similarly, the future of resources to address malnutrition in Addis Ababa may still be misdirected since the traditional interventions to reduce undernutrition cannot also prevent overnutrition. Further quantitative and qualitative research on the evolving socio-economic, cultural, dietary (related to the composition of the urban diet), physical and life style causes are vital to document process of change in overweight/obesity over time. Finally, the co-existence of underweight and overweight/obesity in women of both lower and higher socioeconomic groups in Ethiopia signals the need for further studies focusing on the impact of the socio-economic and demographic transition on the nutrition transition in the country.

In sum, the data presented here unambiguously dictate the increasing threat of overweight or obesity among reproductive-age women, added to the historic prevalence of underweight among younger women in Addis Ababa. The double burden mortality analyses suggest that non-communicable diseases are the leading cause of death among adults in Addis Ababa [22].

In interpreting this study’s findings, it is advisable to consider some of the limitations of the study. The cross-sectional nature of the data doesn’t allow making causal inferences about the relationship between underweight and overweight/obesity and the socioeconomic and demographic correlates. The data also lacks variables on some of the immediate (access to food, caregiver resources) and underlying determinants (cardio-metabolic factors, behaviours and lifestyles) which would be helpful to see their associations with malnutrition. Nevertheless, the study makes use of the most comprehensive dataset in the country at the time.

### Ethical issue

Authorization was obtained from the ICF International to download data from the Demographic and Health surveys (DHS) on-line archive and analyze and present findings.
